# Impact of Serum High Mobility Group Box 1 and Soluble Receptor for Advanced Glycation End-Products on Subclinical Atherosclerosis in Patients with Granulomatosis with Polyangiitis

**DOI:** 10.1371/journal.pone.0096067

**Published:** 2014-04-28

**Authors:** Alexandre W. S. de Souza, Karina de Leeuw, Mirjan M. van Timmeren, Pieter C. Limburg, Coen A. Stegeman, Marc Bijl, Johanna Westra, Cees G. M. Kallenberg

**Affiliations:** 1 Department of Rheumatology and Clinical Immunology, University Medical Center Groningen, University of Groningen, Groningen, The Netherlands; 2 Department of Pathology and Medical Biology, University Medical Center Groningen, University of Groningen, Groningen, The Netherlands; 3 Department of Laboratory Medicine, University Medical Center Groningen, University of Groningen, Groningen, The Netherlands; 4 Department of Internal Medicine, Division of Nephrology, University Medical Center Groningen, University of Groningen, Groningen, The Netherlands; 5 Martini Hospital, Groningen, The Netherlands; 6 Rheumatology Division, Universidade Federal de São Paulo/Escola Paulista de Medicina (Unifesp/EPM), São Paulo-SP, Brazil; University Heart Center Freiburg, Germany

## Abstract

The objective of this study was to evaluate whether levels of high mobility group box 1 (HMGB1) in granulomatosis with polyangiitis (GPA) patients are associated with carotid atherosclerosis, related to levels of soluble receptor for advanced glycation end-products (sRAGE) and influenced by immunosuppressive or lipid-lowering therapy. Twenty-three GPA patients and 20 controls were evaluated for HMGB1- and sRAGE levels and for carotid atherosclerosis using ultrasound to determine intima-media thickness (IMT). *In vitro* the effect of atorvastatin on the production of HMGB1 by lipopolysaccharide (LPS)-stimulated human umbilical vein endothelial cells (HUVEC) was assessed. Serum HMGB1 and sRAGE levels did not differ between patients and controls. A negative correlation was found between sRAGE and maximum IMT but HMGB1 and carotid IMT were not related. HMGB1 levels were reduced in GPA patients on statins and prednisolone. *In vitro*, atorvastatin reduced HMGB1 levels in supernatants of activated HUVEC. In conclusion, carotid IMT is inversely correlated with sRAGE levels but not with HMGB1 levels. Statins and prednisolone are associated with reduced serum HMGB1 levels and atorvastatin decreases HMGB1 release by activated HUVEC *in vitro*, indicating an additional anti-inflammatory effect of statins.

## Introduction

High mobility group box 1 (HMGB1) is a nuclear non-histone DNA binding protein that upon cellular death or activation is released into the extracellular milieu and acts as an alarmin. Extracellular HMGB1 stimulates cytokine production, cell proliferation, chemotaxis, angiogenesis, and cell differentiation through binding to its receptors that include the receptor for advanced glycation end-products (RAGE) and Toll-like receptors (TLR)-2, TLR4 and TLR9 [1]. Increased levels of HMGB1 have been found in patients with granulomatosis with polyangiitis (GPA) with active disease, especially in patients with predominantly granulomatous manifestations and in patients without renal involvement at disease onset [2]–[4]. Individuals with subclinical atherosclerosis and cardiovascular (CV) events present high HMGB1 levels whereas atorvastatin decreases serum HMGB1 in hyperlipidemia [5], [6].

RAGE is a multi-ligand surface molecule involved in the pathogenesis of vascular diseases. Soluble RAGE (sRAGE) is a decoy receptor for RAGE ligands and decreased sRAGE levels are associated with CV events [7]. Patients with GPA present increased advanced glycation end-products (AGEs) accumulation compared to controls and this accumulation is negatively correlated with sRAGE levels [8]. We hypothesized that serum HMGB1 levels contribute to subclinical atherosclerosis in GPA and that this process is influenced by sRAGE but also by treatment with prednisolone and statins.

## Materials and Methods

### Patients and controls

A cross-sectional study was performed on 23 GPA patients and 20 age- and gender-matched controls (Table 1) enrolled in a previous study to evaluate carotid atherosclerosis in GPA [9]. The study was approved by the Medical Ethical Committee (METC) of the University Medical Center Groningen (UMCG) and written informed consent was obtained from patients and controls. GPA was classified according to the European Medicines Agency algorithm [10]. The mean disease duration was 131.2±60.2 months. Birmingham Vasculitis Activity Score (BVAS) was recorded for all patients during admissions and out-patient visits allowing assessment of cumulative BVAS scores from the charts. All GPA patients were evaluated during remission (BVAS = 0) to exclude influence of disease activity. Prednisolone was used by 8 patients (34.8%) at a median of 5 mg/day (3.7–10.0 mg). Statins were prescribed for 5 (21.7%) GPA patients. Healthy controls did not use any medication at the time of the study. Carotid ultrasound was performed to assess intima-media thickness (IMT) and carotid plaques as described previously [9], [11]. Overall mean IMT (mean of IMT measurements performed on carotid bulbus, common and internal carotid arteries) and overall maximum IMT (average of highest IMT values found in above mentioned segments) were used for analysis. Traditional CV risk factors were evaluated according to established guidelines [12]. Serum HMGB1 (Shino Test, Kanagawa, Japan) and sRAGE levels (R&D Systems, Minneapolis, USA) were measured by enzyme-linked immunosorbent assay (ELISA).

**Table 1:** Risk factors for cardiovascular disease, carotid ultrasound, HMGB1 and sRAGE levels in GPA patients and controls.

Variables	GPA patients (N = 23)	Controls (N = 20)	*p*
Median age at study, years	55.2 (45.7–62.4)	49.8 (43.0–57.4)	0.173
Females, n (%)	9 (39.1)	9 (45.0)	0.697
Age >45 years for men and 55 years for women, n (%)	16 (69.6)	12 (60.0)	0.512
Mean total cholesterol, mmol/L	4.99±0.78	4.98±0.82	0.978
Mean HDL, mmol/L	1.41±0.37	1.51±0.33	0.359
Mean LDL, mmol/L	3.01±0.79	3.29±0.82	0.267
Median TGL, mmol/L	1.50 (1.00–2.00)	1.00 (1.00–2.00)	0.404
Mean systolic BP, mmHg	123.65±14.55	119.24±10.94	0.301
Mean diastolic BP, mmHg	70.61±9.38	75.12±8.07	0.120
Smoking, n(%)	2 (8.7)	1 (5.0)	0.635
Family history of premature CVD, n(%)	9 (39.1)	8 (40.0)	0.954
Median BMI, kg/m^2^	26.0 (24.0–28.0)	23.5 (22.0–26.5)	0.256
Previous CVD, n(%)	3 (13.0)	0 (0.0)	0.236
Carotid plaques, n (%)	7 (30.4)	3 (15.0)	0.203
Overall mean IMT, mm	0.833±0.256	0.765±0.133	0.357
Median overall maximum IMT, mm	0.875 (0.810–1.215)	0.880 (0.830–0.990)	0.953
HMGB1, ng/ml	2.13 (1.53–4.15)	2.42 (1.73–4.07)	0.827
sRAGE, pg/mL	1256.1±559.6	1483.3±399.8	0.155

Numerical data are presented as mean ± standard deviation or as median and interquartile range; BMI: body mass index; BP: blood pressure; CVD: cardiovascular disease; HDL: high-density lipoprotein; HMGB1: high mobility group box-1; IMT: intima-media thickness; LDL: low-density lipoprotein; n: number of individuals; sRAGE: soluble receptor for advanced glycation end-products; TGL: triglycerides.

### Cell cultures

HUVEC (Lonza, Breda, The Netherlands) were cultured in EBM-2 medium supplemented with EGM-2 MV Single Quot Kit Supplements & Growth Factors (cat No. CC-3202, Lonza) and used when confluent. Three groups were evaluated: (1) HUVEC pre-incubated for 2 hours with 5 µM atorvastatin (Sigma Aldrich, Saint Louis, USA) and treated with LPS (100 ng/ml) (Sigma Aldrich, Saint Louis, USA), (2) HUVEC treated only with LPS, and (3) unstimulated HUVEC. Supernatants were collected for measuring HMGB1 and interleukin (IL)-8 at baseline, 4 hours and 24 hours. All *in vitro* experiments were performed twice and in duplicate. Cell viability of HUVEC was checked with 0.2% trypan blue dye (Invitrogen, Carlsbad, USA) on HUVEC treated with 0 µM, 0.1 µM, 1.0 µM, 5.0 µM and 10.0 µM atorvastatin; percentages of living cells were 94%, 95%, 95%, 92% and 91% at 36 hours, respectively. Western blot was used to measure HMGB1 in supernatants as previously described [13] while IL-8 levels were measured by ELISA (R&D Systems, Minneapolis, USA).

### Statistical analysis

Statistical analysis was performed with SPSS 18.0 software and graphs were built using GraphPad Prism 5. Continuous variables are presented as mean ± SD or as median and interquartile range. Categorical variables are presented as total number and percentage. Comparisons between groups were performed using chi-square test or Fisher's exact test and Student's t-test or Mann-Whitney U test as appropriate. Analysis of longitudinal data from *in vitro* experiments was performed by two-way ANOVA and Bonferroni's test. Correlations were evaluated with Spearman's rank correlation coefficient. Differences were considered significant when *p*<0.05.

## Results

### HMGB1, sRAGE and subclinical atherosclerosis

**Figure 1:**
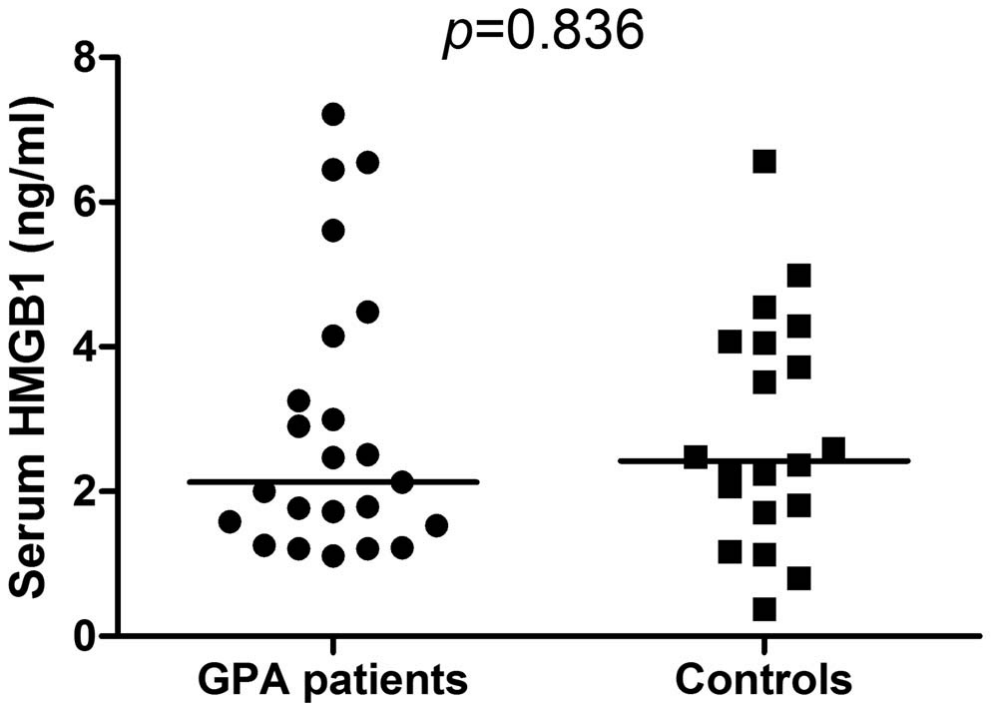
Serum HMGB1 levels in GPA patients and controls. GPA patients in remission present similar median HMGB1 levels compared to controls.

**Figure 2:**
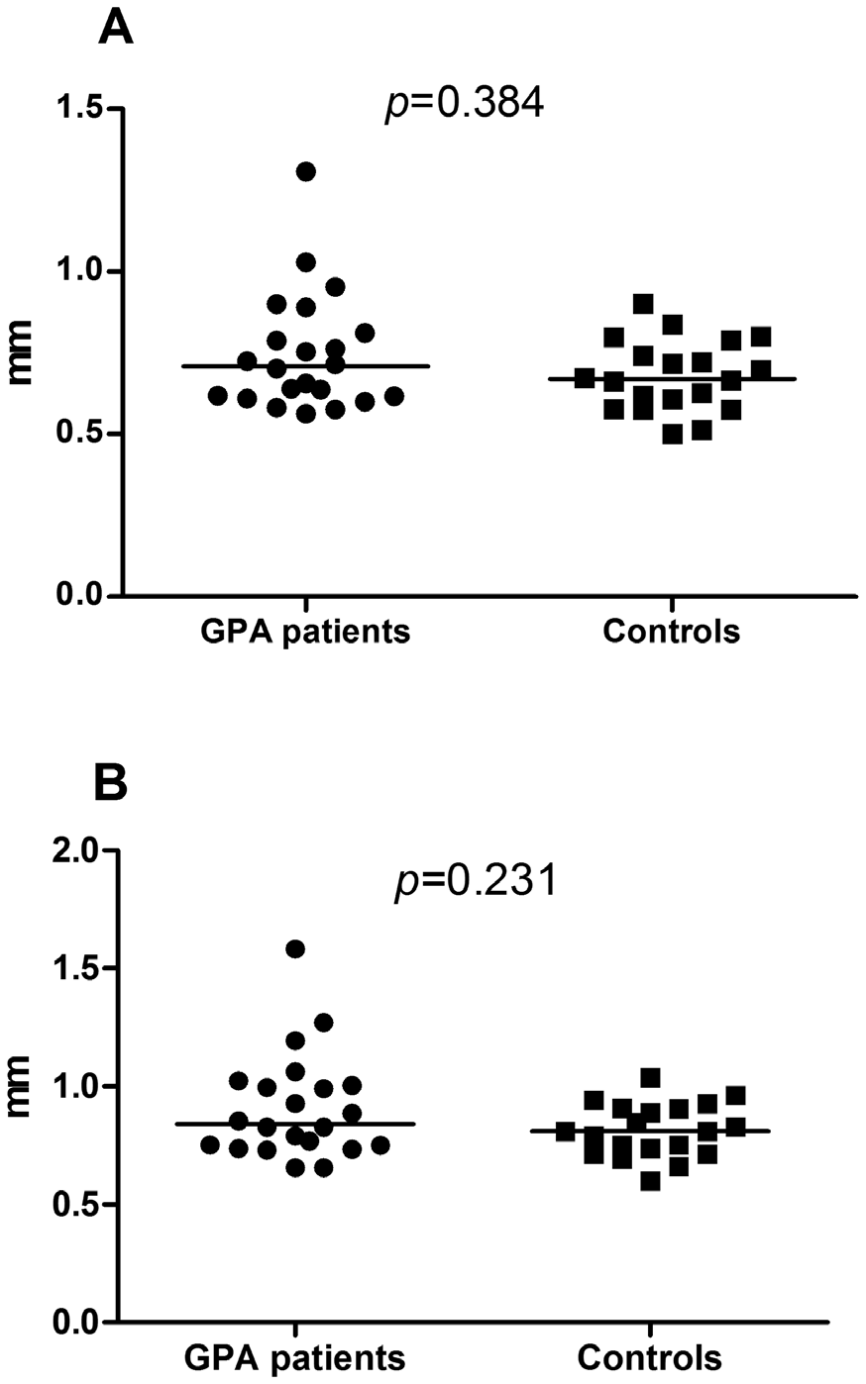
Intima media thickness in common carotid arteries in GPA and controls. GPA patients present mean IMT (2A) and maximum IMT (2B) in common carotid arteries similar to controls.

Figure 1 shows similar serum HMGB1 levels in patients and controls whereas Figures 2A and 2B depicts similar overall mean IMT and maximum IMT in carotid arteries in GPA and controls. Furthermore, sRAGE levels and the prevalence of carotid plaques did not differ between patients and controls (Table 1). In GPA patients, no correlation was found between HMGB1 and overall maximum IMT in carotid arteries (rho = 0.062; *p* = 0.820). However, sRAGE levels were negatively correlated with overall maximum IMT in carotid arteries (rho = −0.565; *p* = 0.035). No correlation was present between HMGB1 and sRAGE (rho = 0.068; *p* = 0.777), between overall mean carotid IMT and cumulative BVAS (rho = −0.070; *p* = 0.805) or between overall mean carotid IMT and time in remission since last relapse/presentation (rho = 0.337; *p* = 0.201). Moreover, no correlation was found between overall maximum carotid IMT and cumulative BVAS (rho = −0.050; *p* = 0.859) and between overall maximum IMT and time in remission since last relapse/presentation (rho = 0.338; *p* = 0.200). Also, no significant difference was found between patients with and without carotid plaques regarding HMGB1 [1.52 (1.20–2.89) ng/ml vs. 2.48 (1.74–4.22) ng/ml; *p* = 0.300] and sRAGE levels (1144.66±817.33 pg/ml vs. 1303.99±438.86 pg/ml; *p* = 0.574).

### Impact of treatment on HMGB1 and sRAGE

**Figure 3:**
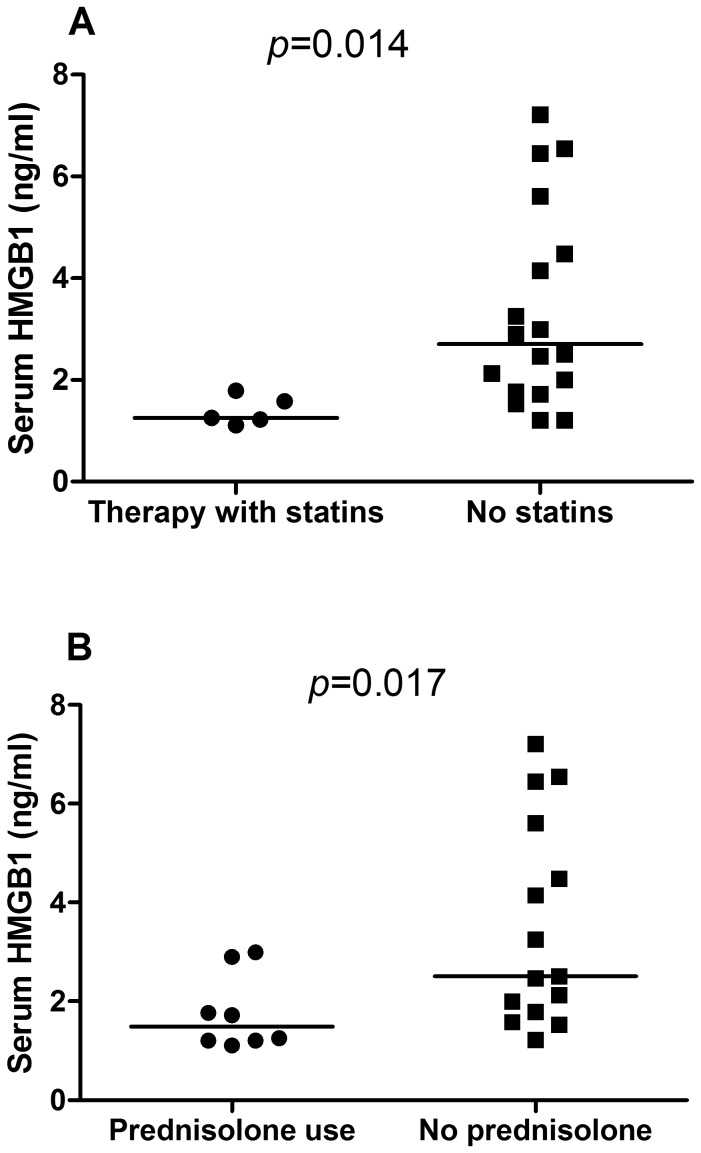
HMGB1 levels in GPA patients treated with statins or prednisolone. median serum levels of HMGB1 were significantly lower in GPA patients treated with statins (3A) and prednisolone (3B) when compared to patients without these drugs.

Since HMGB1 and sRAGE levels did not differ between GPA patients in remission and controls, we evaluated whether therapy would have any influence on both biomarkers. Serum HMGB1 levels were lower in patients on statins [1.26 (1.16–1.68) ng/ml vs. 2.70 (1.75–4.76) ng/ml; *p* = 0.014] and on prednisolone [1.49 (1.21–2.61) ng/ml vs. 2.51 (1.79–5.61) ng/ml; *p* = 0.017] compared to patients without these drugs, respectively (Figure 3A and 3B). Although not significant, we observed higher serum HMGB1 levels in 12 GPA patients without statins or prednisolone compared with 20 controls (4.03±2.00 ng/ml vs. 2.84±1.58 ng/ml, *p* = 0.073). sRAGE levels did not differ between patients with and without statins (976.20±345.64 pg/ml vs.1326.19±588.79 pg/ml; *p* = 0.275) or prednisolone (1366.96±450.80 pg/ml vs. 1196.55±619.08 pg/ml; *p* = 0.531).

### HMGB1 and atorvastatin in HUVEC

**Figure 4:**
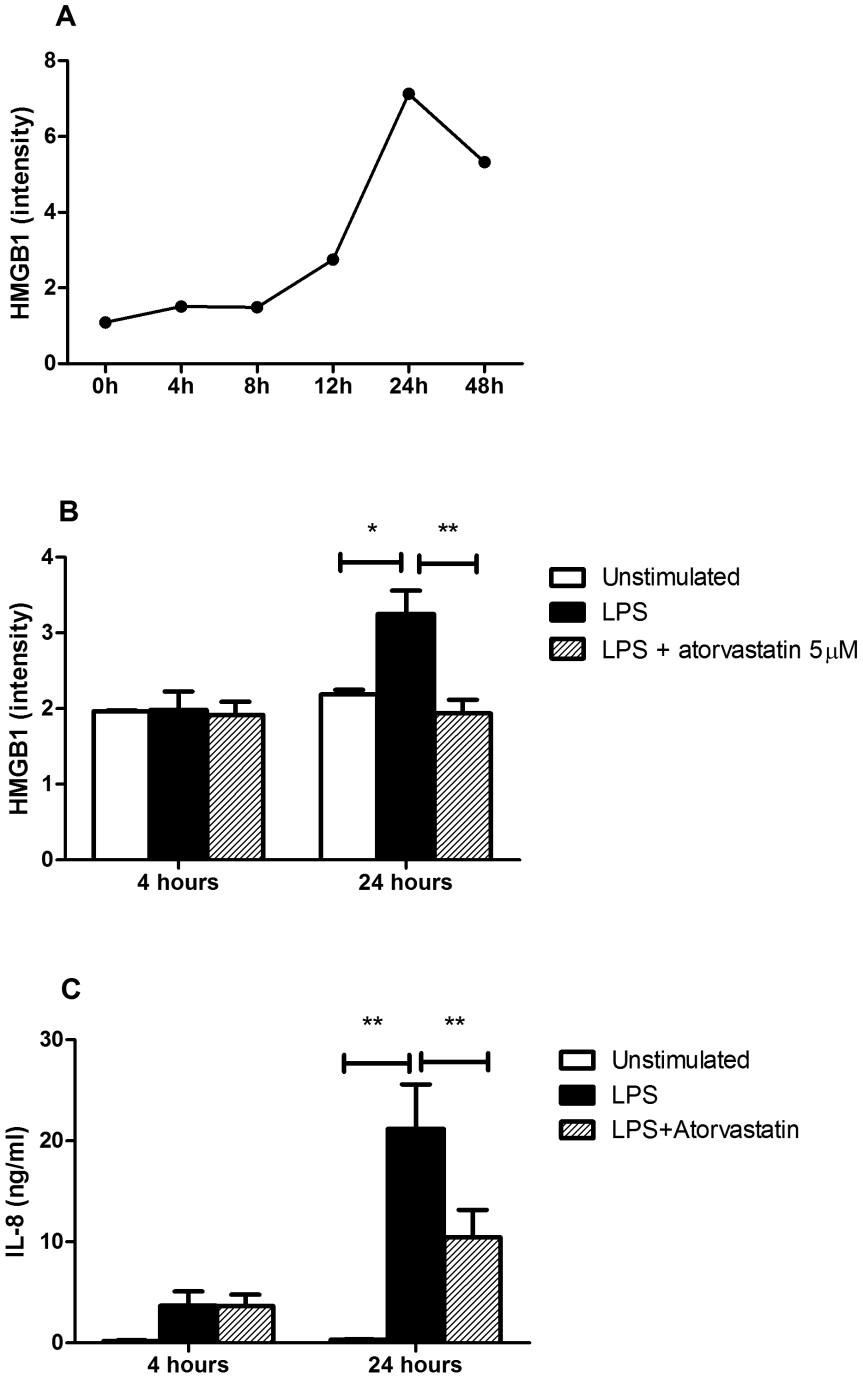
Effect of atorvastatin on HMGB1 and IL-8 levels in HUVEC supernatants. 4A – A time curve was built to evaluate HMGB1 release from HUVEC upon LPS stimulation. HMGB1 intensity in supernatants increases 8 hours after stimulation and a peak is observed at 24 hours with a decrease at 48 hours. 2B and C – HMGB1 and IL-8 levels are significantly higher at 24 hours in supernatants from HUVEC treated with LPS in comparison to unstimulated HUVEC while pre-incubation with 5 µM atorvastatin followed by LPS stimulation lowered HMGB1 and IL-8 levels significantly at 24 hours in HUVEC's supernatants. Experiments were performed twice and in duplicate. Data are presented as median and range, *p<0.01 and **p<0.001.

To investigate the relation between statins and HMGB1, we tested the effect of atorvastatin on HMGB1-release from LPS-stimulated HUVEC. HMGB1 in supernatants gradually increased in time with a peak after 24 hours of LPS stimulation and a decrease at 48 hours (Figure 4A). Thus, we compared HMGB1 and IL-8 levels in supernatants of HUVEC pre-incubated with atorvastatin followed by 24 hours of LPS stimulation. HUVEC stimulated with LPS showed a significantly higher production of HMGB1 and IL-8 than unstimulated cells. Pre-incubation with 5 µM atorvastatin prior to LPS stimulation led to significantly lower levels of both HMGB1 and IL-8 in supernatants after 24 hours in comparison to HUVEC stimulated by LPS only (Figures 4B and 4C).

## Discussion

In this study, in GPA patients HMGB1 levels were not correlated with overall carotid maximum IMT, whereas sRAGE levels were negatively correlated with IMT. Furthermore, statins or prednisolone use was associated with lower HMGB1 levels.

In previous studies, GPA patients presented higher IMT levels in carotid arteries and an increased number of CV events compared to controls [11], [14]. Accelerated atherosclerosis in GPA was not associated with traditional CV risk factors, rather enhanced levels of markers of vascular inflammation and remodeling were associated with atherosclerotic disease [11]. In this study, we evaluated HMGB1 and sRAGE levels in GPA patients who had been enrolled in a follow-up study to evaluate progression of atherosclerosis [9]. Patients were in remission and a reduction of traditional CV risk factors was achieved during follow-up of these patients that possibly resulted in similar IMT and similar prevalence of carotid plaques compared to controls [9]. Also, cumulative BVAS scores and time in remission were not related to IMT values. Moreover, proteinase 3 antineutrophil cytoplasmic antibody (PR3-ANCA) status has been shown to have a protective role on the risk of CV events in patients with ANCA-associated vasculitis and most of our patients were PR3-ANCA positive [15].

HMGB1 and sRAGE have been implicated in the pathogenesis of atherosclerotic disease. Higher serum HMGB1 levels are a potential marker of subclinical atherosclerosis and CV events while lower serum sRAGE levels are associated with risk factors for CV disease and CV events [5], [7]. Thus, we evaluated the association between both biomarkers and atherosclerotic disease in GPA. sRAGE was associated with subclinical carotid atherosclerosis whereas HMGB1 was not. We hypothesized that due to its multifaceted nature, HMGB1 is possibly more influenced by other factors such as therapy while sRAGE levels are more stable. Indeed, prednisolone or statins use were associated with lower serum HMGB1 levels in our study.

Previous studies had also shown reduction of HMGB1 levels upon statins in an experimental model of atherosclerosis and in humans with hyperlipidemia [6], [16]. In this study, we demonstrated that statins are associated with lower serum HMGB1 levels in GPA patients in remission while a tendency for higher serum HMGB1 levels was observed in GPA patients in remission without statins compared with controls. Moreover, the addition of 5 µM atorvastatin which is within the range of atorvastatin concentration used in other in vitro studies [17]–[21] led to a decrease in extracellular HMGB1 levels in activated HUVEC. These findings indicate that endothelial cells are a possible source of extracellular HMGB1 that could be inhibited by atorvastatin. The decrease in IL-8 levels in supernatants of activated HUVEC by atorvastatin was used as a comparator since it is already known that statins inhibit mRNA expression of IL-8 in activated HUVEC [22]. Inhibition of HMGB1 release by activated HUVEC points to another potential anti-inflammatory effect of statins on vascular endothelium. The actual mechanism of inhibition of HMGB1 release by HUVEC (i.e. inhibition of mRNA expression or cytoplasmic translocation of HMGB1) still needs further elucidation.

HMGB1 levels were also lower in GPA patients on prednisolone therapy. Previous studies have demonstrated that corticosteroids can reduce extracellular release of HMGB1 by monocytes *in vitro* and they can also reduce HMGB1 expression and circulating levels *in vivo* [23], [24]. However, these findings were not confirmed in patients with rheumatoid arthritis [25].

In conclusion, no association was observed between subclinical carotid atherosclerosis and HMGB1 while sRAGE was negatively associated with carotid IMT in GPA. Statin use was associated with lower HMGB1 levels suggesting an additional anti-inflammatory property of statins. As subclinical atherosclerosis was similar between patients and controls, we suggest that development of premature atherosclerosis in GPA patients might be postponed by sRAGE and use of statins or prednisolone. Since our study had a relatively low number of patients and had a cross-sectional design, further longitudinal studies are needed to evaluate if reduction of serum HMGB1 levels might be important in CV risk management in GPA.
